# Electrochemical Biosensor for Rapid and Sensitive Detection of Magnetically Extracted Bacterial Pathogens

**DOI:** 10.3390/bios2010015

**Published:** 2012-01-17

**Authors:** Emma B. Setterington, Evangelyn C. Alocilja

**Affiliations:** Department of Biosystems and Agricultural Engineering, Michigan State University, East Lansing, MI 48824, USA; E-Mail: ebs@msu.edu

**Keywords:** electrochemical biosensor, pathogen detection, magnetic polyaniline, screen-printed carbon electrode, cyclic voltammetry

## Abstract

Biological defense and security applications demand rapid, sensitive detection of bacterial pathogens. This work presents a novel qualitative electrochemical detection technique which is applied to two representative bacterial pathogens, *Bacillus cereus* (as a surrogate for *B. anthracis*) and *Escherichia coli* O157:H7, resulting in detection limits of 40 CFU/mL and 6 CFU/mL, respectively, from pure culture. Cyclic voltammetry is combined with immunomagnetic separation in a rapid method requiring approximately 1 h for presumptive positive/negative results. An immunofunctionalized magnetic/polyaniline core/shell nano-particle (c/sNP) is employed to extract target cells from the sample solution and magnetically position them on a screen-printed carbon electrode (SPCE) sensor. The presence of target cells significantly inhibits current flow between the electrically active c/sNPs and SPCE. This method has the potential to be adapted for a wide variety of target organisms and sample matrices, and to become a fully portable system for routine monitoring or emergency detection of bacterial pathogens.

## 1. Introduction

Microbial pathogens are some of the deadliest “enemies” encountered in biodefense and biosecurity. Standard culture methods for identifying pathogenic bacteria cannot provide results fast enough to be useful in an emergency situation such as a biological attack. There is a tremendous need for rapid and sensitive methods of bacterial detection for defense and security applications. This work presents a novel electrochemical detection technique which is applied to two representative bacterial pathogens, *Bacillus cereus* and *Escherichia coli* O157:H7. Detection is combined with a previously published method [1,2] of immunomagnetic extraction, making the technique applicable to a wide variety of target organisms and sample matrices.

*B. cereus*, a gram-positive bacterium, is a common foodborne pathogen able to cause diarrhea and abdominal pain with an infectious dose of about 10^6^ colony-forming units (CFU) per gram of food [3,4]. Although the high infectious dose and non-fatal symptoms make this bacteria an unlikely candidate for a biological weapon, *B. cereus* is phenotypically very similar to *Bacillus anthracis*. This study utilizes *B. cereus* as a surrogate (model) organism for *B. anthracis*, the causative agent of anthrax. The Centers for Disease Control and Prevention (CDC) and the National Institute of Allergy and Infectious Diseases (NIAID) classify *B. anthracis* as a “Category A” priority pathogen in biodefense [5,6]. *B. anthracis* spores can withstand harsh conditions, including heat, radiation, and chemicals, for long periods of time and remain infectious, making the organism an ideal agent for biological warfare [2,4], An unknown microorganism can be identified as a *Bacillus* species by standard culture methods and biochemical tests within 24 h, but definitive identification of *B. anthracis* may require another 1–2 days [2,4].

*E. coli* O157:H7, a gram-negative bacterium and a type of enterohemorrhagic *E. coli* (EHEC), is a potentially fatal food and water borne pathogen, with an infectious dose of 10 to 100 CFU [7]. Symptoms of infection with *E. coli* O157:H7 include abdominal cramps, bloody diarrhea, nausea, vomiting, headache, and (in 2–7% of cases) life-threatening hemolytic uremic syndrome (HUS), characterized by kidney failure and hemolytic anemia [8]. The pathogen is a fecal contaminant commonly found in raw or undercooked meat, unwashed produce, unpasteurized milk, and sewage-tainted waters. The United States Centers for Disease Control and Prevention (CDC) and the National Institute of Allergy and Infectious Diseases (NIAID) classify *E. coli* O157:H7 as a “Category B” (second-highest priority) pathogen for biodefense, because of its ease of dissemination in water and food sources [5,6]. Additionally, the CDC cites at least nine confirmed food-linked outbreaks of *E. coli* O157:H7 infection in the U.S. from 2006 to 2009 [9] and estimates that 265,000 Shiga toxin-producing *E. coli* infections occur each year in the U.S. alone (36% of these are caused by O157:H7) [10]. This indicates a vital need for improved surveillance, diagnostic methodologies, prevention strategies, and food and water monitoring techniques. 

The standard method of identifying *E. coli* O157:H7 from unknown samples requires 2 to 3 days, and involves enrichment in selective media followed by growth on differential agar to isolate sorbitol non-fermenting colonies. These are identified phenotypically and serologically, and toxigenically characterized by PCR. The standard method is able to detect as few as 1 CFU/g in foods [7]. 

Since standard methods of identifying both *E. coli* O157:H7 and *B. cereus* or *B. anthracis* require at least a full day (to rule out a negative sample) and up to several days (to confirm a positive result), there has been much research directed toward developing rapid detection methods for these organisms. The most practical and field-ready detection methods begin with extraction, purification, and concentration of target cells, in order to eliminate lengthy enrichment steps and interference from the sample matrix during detection. One efficient extraction and concentration method is immunomagnetic separation (IMS). In IMS, micro- or nanometer scale magnetic particles are immunofunctionalized with antibody, incubated with the sample to bind target cells, and separated from the sample matrix through application of a magnetic field. The magnetic particle-bound target can then be washed and suspended at a higher concentration in the testing medium. In comparison to centrifugation, filtration, or capture of target on an immunofunctionalized surface, IMS is simpler and generally results in higher capture efficiency due to the greater surface area available for target binding. IMS has been paired with a variety of rapid detection methods for bacterial pathogens [11], including fluorescence and bioluminescence techniques [12,13,14,15] and enzymatic assays [16,17,18]. Only a few methodologies have been reported which combine IMS with label-free impedance-based bacterial detection [19,20,21]. 

Electrochemical transducers have emerged as an excellent choice for biosensor applications due to their low cost, miniaturization, and potential portability. In general, they require simpler equipment, are more easily integrated with electronic readout devices, and are less susceptible to environmental effects and contaminants than other analytical techniques [22]. Traditional electrochemistry is performed in solution in a three-electrode electrochemical cell. In this system, the *working electrode* is the site at which current is measured. A potential is applied to this electrode relative to a second *reference electrode*, also in contact with the test solution. The *counter electrode* is part of a feedback system which supplies current to the test solution when necessary in order to maintain the correct potential at the working electrode-solution interface [23]. Cyclic voltammetry is a common electrochemical measurement technique in which a cycling electric potential is applied between the electrodes and the resulting current flow is measured.

An important advance in biosensor technology has been the miniaturization of electrochemical systems, which reduces sample and reagent volumes, reduces response time due to faster diffusion, makes the sensor more economical, and is more conducive to portability. Most electrochemical biosensors today use microelectrodes (micrometer to millimeter scale) on compact sensing chips to perform electrochemistry on a small scale. The “electrochemical cell” in these miniaturized systems is simply a reaction well in contact with two electrodes (the reference and counter electrodes are usually combined into one in miniaturized systems). The small sensing chips often consist of screen-printed electrodes on a glass or plastic substrate. Screen-printing involves deposition of metallic or semiconductive ink to form electrodes of a specific shape and size, with constant thickness. Screen-printed electrode sensors can be mass-produced, inexpensive, and disposable, which avoids the possibility of electrode fouling and cross-contamination between samples due to reuse of electrodes [22]. Screen-printed carbon [24,25] and gold [26,27,28] electrodes have been recently applied to impedance-based detection of *E. coli* and other bacterial targets. 

Conducting polymers have found application in electrochemical biosensors since they are electrically active, compatible with biological molecules, simple to synthesize, and environmentally stable [29]. One of the most studied and well characterized conducting polymers is polyaniline, because its different physical, chemical, and electronic states can be controlled and exploited for various purposes. Polyaniline can be switched from the electrically insulating (semiconducting, emeraldine base) form to the conducting (metallic, emeraldine salt) form by doping with a protonic acid, and can be converted back to the insulating form by addition of a base [30]. Furthermore, polyaniline has well-defined, reversible redox chemistry, and is easily recognized in cyclic voltammetry by its characteristic oxidation and reduction peaks [31]. 

The development of a novel magnetic and conductive nanoparticle for both IMS and electrical transducing applications in biosensors has been previously reported by this laboratory [1,2]. The nanoparticle consists of an iron oxide core, imparting magnetic functionality, and a polyaniline shell, imparting electronic activity. In this work, the immunofunctionalized magnetic/polyaniline core/shell nanoparticle (c/sNP) is employed for IMS, for magnetic positioning of target bacterial cells on a screen-printed carbon electrode (SPCE) sensor, and as a mediator for current flow. A new electrochemical detection technique is presented, in which the presence of c/sNP-bound target cells inhibits current response in cyclic voltammetry. The technique enables rapid detection of *B. cereus* and *E. coli* O157:H7 at low concentrations (40 CFU/mL and 6 CFU/mL, respectively). The versatility of IMS makes this method applicable to a wide variety of target organisms and sample matrices. The system could potentially become fully portable and be deployed in government facilities, military zones, airports and public transit, or food and water supply centers, for routine monitoring or emergency detection of bacterial pathogens.

## 2. Materials and Methods

### 2.1. Reagents and Materials

Iron (III) oxide (γ-Fe_2_O_3_) nanopowder, aniline monomer, hydrochloric acid (HCl), ammonium persulfate, methanol, and diethyl ether were used for synthesis of core/shell nanoparticles. Polysorbate-20 (Tween-20), phosphate buffered saline (PBS), trizma base, casein from bovine milk, and sodium phosphate (dibasic and monobasic) were used in conjugation of antibodies to nanoparticles and in capture of bacterial cells from culture media. All of the above reagents were purchased from Sigma-Aldrich (St. Louis, MO, USA). Potassium ferricyanide (Spectrum Chemical Mfg. Corp, Gardena, CA, USA) was used for validation of SPCE electroactivity. 

Polyclonal anti-*Bacillus* antibodies and monoclonal anti-*E. coli* O157:H7 antibodies were obtained from Meridian Life Science, Inc. (Saco, ME, USA). *Bacillus cereus* strain R4 and a strain of *E. coli* O157:H7 were obtained from the collection of the Nano-Biosensors Laboratory at Michigan State University, and grown in tryptic soy broth (BD Biosciences, MD, USA) at 37 °C for 24 h. Cells were serially diluted in 0.1% w/v peptone water (Fluka-Biochemika, Switzerland) prior to detection. Viable cells were enumerated by microbial plating (20 h incubation at 37 °C) on trypticase soy agar II (BD Biosciences, MD, USA).

All solutions and buffers used in this study were prepared in de-ionized (DI) water (from Millipore Direct-Q system) as follows: phosphate buffer (100 mM sodium phosphate, pH 7.4), blocking buffer (100 mM Tris-HCl buffer, pH 7.6, with 0.01% w/v casein), PBS buffer (10 mM PBS, pH 7.4), and wash buffer (10 mM PBS, pH 7.4, with 0.05% Tween-20). Magnetic separations were performed with a commercial magnetic separator (FlexiMag, SpheroTech Inc., IL, USA). Hybridization reactions were carried out on a rotisserie-style tube rotator (Labquake, Thermo Fisher Scientific Inc., MA, USA).

### 2.2. Apparatus

**Figure 1:**
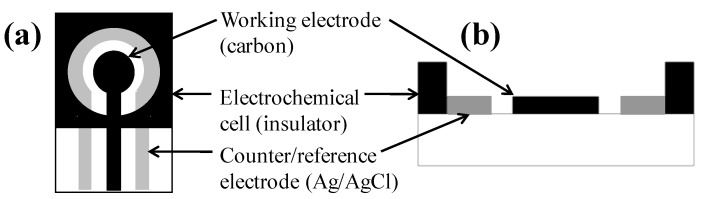
Schematic of the SPCE sensor: (**a**) top view; (**b**) cross-sectional view.

Cyclic voltammetric measurements were performed with a 263A potentiostat/galvanostat (Princeton Applied Research, MA, USA) connected to a personal computer. Data collection and analysis was controlled with the PowerSuite electrochemical software operating system (Princeton Applied Research, MA, USA). Screen-printed carbon electrode (SPCE) sensors (Gwent Inc., UK) are shown in Figure 1. The sensor is composed of two electrodes: the inner carbon/graphite working electrode, having a diameter of 4 mm, and the outer silver/silver chloride counter/reference electrode. A 200 μL capacity sample well is defined by an insulating foam. Every SPCE sensor was rinsed with sterile DI water and allowed to dry before test solution was applied. Sensors were disposed of after a single use.

### 2.3. Preparation of Pure Polyaniline Nanoparticles

Nanoparticles of pure polyaniline (non-magnetic) were synthesized and electrochemically characterized for comparison with magnetic/polyaniline c/sNPs. Pure polyaniline nanoparticles were produced by chemical polymerization and acid doping of aniline monomer [32,33,34]. A volume of 3 mL of 10 M HCl and 3 mL of aniline were suspended in DI water and cooled to 0 °C. Ammonium persulfate (7.5 g) in aqueous solution was incrementally added to the aniline solution, and the reaction continued with constant stirring at 0 °C for 4 h. Finally the solution was filtered, washed with DI water and 10% methanol, and dried for 18 h. The resulting solid was ground into fine powder and stored in a vacuum desiccator.

### 2.4. Preparation and Characterization of Core/Shell Nanoparticles

Magnetic/polyaniline core/shell nanoparticles (c/sNPs) were synthesized by polymerization and acid doping of aniline monomer around gamma iron (III) oxide (γ-Fe_2_O_3_) nanoparticles [35]. Briefly, commercial γ-Fe_2_O_3_ nanoparticles were dispersed in 50 mL of 1 M HCl, 10 mL DI water, and 0.4 mL aniline monomer by sonication at 0 °C for 1 h. The γ-Fe_2_O_3_ to monomer weight ratio was fixed at 1:0.6. Oxidant (1 g ammonium persulfate in 20 mL DI water) was added drop-wise while the mixture was stirred at 0 °C. Color change from rust brown to dark green indicated formation of electrically-active (green) polyaniline over the smaller (brown) γ-Fe_2_O_3_ nanoparticles. The reaction continued with constant stirring at 0 °C for 4 h. Finally the solution was filtered, washed with 1 M HCl, 10% methanol, and diethyl ether, and dried for 18 h. The resulting solid was ground into fine powder and stored in a vacuum desiccator. 

These electrically-active magnetic/polyaniline c/sNPs have been previously characterized and compared with unmodified γ-Fe_2_O_3_ nanoparticles in [1,2]. A 200 kV field emission transmission electron microscope (JEOL 2200 FS) was used to investigate structural properties and size distribution. A Four Point Probe (Lucas/Signaton Corp., Pro4, CA, USA) was used to measure solid state electrical conductivity at room temperature. Magnetic properties were evaluated with a superconducting quantum interference device (Quantum Design MPMS SQUID) magnetometer. Hysteresis magnetization was measured with field cycling between +15 and –15 kOe, at a constant temperature of 300 K.

### 2.5. Electrochemical Characterization of Nanoparticles

Cyclic voltammetric tests were performed for electrochemical characterization of pure (nonmagnetic) polyaniline nanoparticles, magnetic/polyaniline c/sNPs, and immuno-c/sNPs, each suspended in 0.1 M HCl or in PBS buffer. The acidic solution re-dopes the polyaniline producing a color change from blue back to green. A volume of 100 μL of the appropriate nanoparticle solution was placed in the electrochemical cell of an SPCE sensor fixed onto a magnetic surface, and cyclic voltammetry was performed between −0.4 V and +1.0 V at 50 mV/s. Each test consisted of three consecutive voltammetric cycles, and for each cycle the response current data and the value of total charge transfer (ΔQ) were recorded. 

### 2.6. Immunomagnetic Separation of Bacterial Target

The electrically active c/sNPs were conjugated with antibodies by direct physical adsorption as described and confirmed in [2]. Polyclonal anti-*Bacillus* antibodies (50 μg/mL) were mixed with c/sNPs (10 mg/mL) in phosphate buffer and hybridized for 1 h at room temperature with rotation. The bio-modified particles (immuno-c/sNPs) were magnetically separated to remove any unbound antibody in the supernatant, washed twice with blocking buffer, resuspended in phosphate buffer, and stored at 4 °C. 

**Figure 2:**
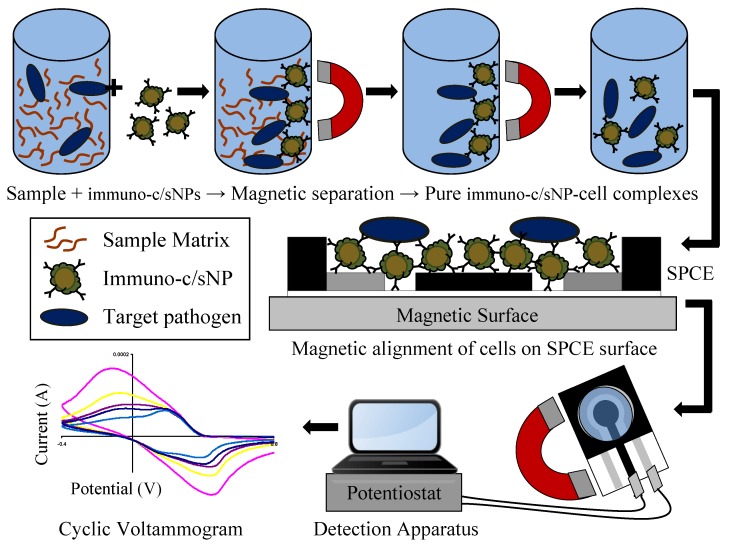
Immunomagnetic separation from sample matrix, magnetic alignment on SPCE surface, and electrochemical detection of *B. cereus* and *E. coli* O157:H7 cells.

The immuno-c/sNPs were used to isolate target cells from pure culture. Serial dilutions of *B. cereus* and *E. coli* O157:H7 cultures grown overnight in trypticase soy agar were prepared independently of one another in 0.1% (w/v) peptone water. Immuno-c/sNPs and an aliquot of the appropriate bacterial dilution (containing serially diluted cells and culture media) were combined in PBS buffer (for a final c/sNP concentration of 1 mg/mL), and hybridized for 30 min at room temperature with rotation. The immuno-c/sNP-cell complexes were magnetically separated and the supernatant removed. Complexes were washed twice with wash buffer (Tween-20 in PBS) and resuspended in PBS buffer. The immuno-c/sNP-cell solutions, and also pure bacterial dilutions, were plated to determine the number of viable cells present. Capture efficiency was calculated as the number of viable cells captured divided by the number of viable cells in the original dilution. The immunomagnetic separation procedure is depicted in Figure 2. Upon completion of immuno-functionalization of c/sNPs and immunomagnetic separation of target cells, the polyaniline shell of the c/sNPs visibly changed color from dark green to dark blue. 

### 2.7. Electrochemical Detection of Bacterial Target

The immuno-c/sNP-cell complexes were magnetically separated from the supernatant (PBS buffer) and re-suspended in 0.1 M HCl for 20 min (optimized time, data not shown), in order to reactivate the polyaniline by acid doping. Upon addition of the acid, the c/sNPs underwent a color change from blue back to green. 

The electrochemical detection apparatus is depicted in Figure 2. Immediately following the 20 min incubation period, a volume of 100 μL of the immuno-c/sNP-cell complexes in 0.1 M HCl was added to the sample well of the SPCE sensor, and the sensor was placed on a magnetic platform in order to attract and orient the immuno-c/sNP-cell complexes tightly onto the sensor surface (Figure 2), where the electrochemical effect of the cells is maximized. The SPCE sensor was connected to the potentiostat/galvanostat with specially adapted electrical connector clips, and a voltammetric cycle between −0.4 V and +1.0 V was applied at a scan rate of 50 mV/s. Each sensor was scanned with three complete, consecutive cycles, and the response current data and the value of total charge transfer (ΔQ) were recorded. Cyclic voltammetric tests were performed in the same way for pure *B. cereus* and *E. coli* O157:H7 cells (in the absence of c/sNPs) suspended in 0.1 M HCl.

## 3. Results and Discussion

### 3.1. Preparation and Characterization of Core/Shell Nanoparticles

Characterization studies [2] of the magnetic/polyaniline c/sNPs indicate a value of 3.3 S/cm in room temperature solid state electrical conductivity, whereas unmodified γ-Fe_2_O_3_ nanoparticles show a conductivity of 3.4 × 10^−5^ S/cm. The high conductivity of the synthesized c/sNPs confirms the presence of electrically-active polyaniline. Both the c/sNPs and the unmodified γ-Fe_2_O_3_ nanoparticles demonstrate superparamagnetic behavior, meaning they only become magnetic when exposed to an external magnetic field. At an applied field of 15 kOe, c/sNPs display a room temperature saturation magnetization value of 44.1 emu/g. Although lower than the saturation magnetization of unmodified γ-Fe_2_O_3_ nanoparticles (64.4 emu/g under the same conditions), the magnetization of the c/sNPs is more than sufficient for the intended applications of IMS and magnetic positioning of target cells. Transmission electron microscopy studies indicate an average diameter of 20 nm for unmodified γ-Fe_2_O_3_ nanoparticles and a range in diameter from 50 to 100 nm in synthesized magnetic/polyaniline c/sNPs.

### 3.2. Electrochemical Characterization of Nanoparticles

**Figure 3:**
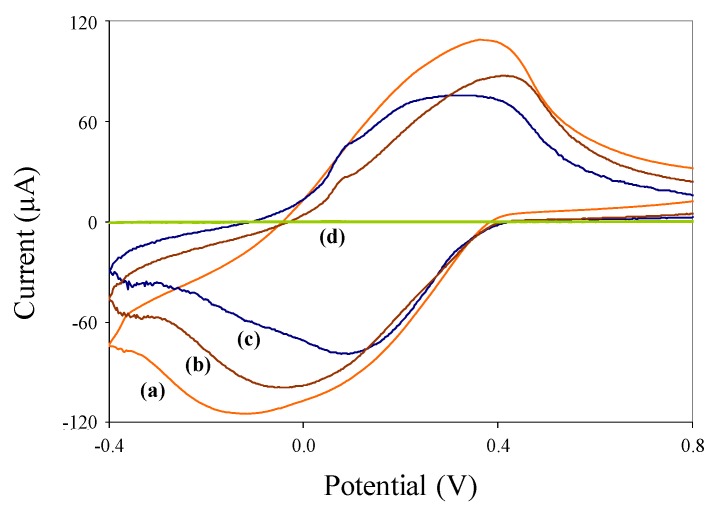
Cyclic voltammograms of (**a**) 1 mg/mL c/sNPs; (**b**) 1 mg/mL immuno-c/sNPs; (**c**) 0.1 mg/mL polyaniline nanoparticles, each suspended in 0.1 M HCl solution; and (**d**) 0.1 M HCl solution alone.

Cyclic voltammograms of pure (nonmagnetic) polyaniline nanoparticles, synthesized c/sNPs, and immuno-c/sNPs, each in 0.1 M HCl solution, are shown in Figure 3. The same tests performed in PBS buffer resulted in much lower current responses (data not shown), due to lack of polyaniline doping.

The HCl solution alone produces a very low background signal (curve d). The presence of polyaniline nanoparticles (curve c) results in a characteristically-shaped voltammogram for pure electroactive polyaniline at slightly acidic pH values [31]. Pure polyaniline nanoparticles were tested at a lower concentration (0.1 mg/mL) than c/sNPs (1 mg/mL) for ease of display in a single figure. The c/sNPs (curve a) also show the characteristic electroactive polyaniline shape, although a higher concentration (1 mg/mL) of particles is needed to produce a current response with approximately the same magnitude as that of pure polyaniline (at 0.1 mg/mL). This is to be expected, since the greater portion of the c/sNP mass is iron oxide, not polyaniline (weight ratio of iron oxide to aniline monomer during c/sNP synthesis is 1:0.6). Immuno-functionalization slightly decreases the electroactivity of the c/sNPs, as shown by the attenuated current response in curve b. This is likely due to the immobilized antibodies and casein blocking protein inhibiting electron transfer at the polyaniline surface.

### 3.3. Immunomagnetic Separation of Bacterial Target

The green color of the synthesized c/sNPs indicates the presence of the electroactive (protonated) form of polyaniline, which is expected to result from the acidic chemical polymerization method employed here. Throughout the immunofunctionalization and target extraction procedures, which were carried out in slightly basic conditions (pH 7.4–7.6), the shell of the c/sNPs became at least partially de-protonated to the emeraldine base form of polyaniline, as evidenced by the color change from green to blue. This pH-induced redox reversibility is a well-characterized property of polyaniline [31,36]. Incubation in HCl and the accompanied color change from blue back to green ensures that the polyaniline is fully doped and conducting prior to electrochemical tests.

Immunofunctionalization of c/sNPs was carried out by physical adsorption of antibodies onto the polymer shell. Electrostatic interactions between the negatively charged Fc portion of the antibodies and the positively charged polyaniline surface are thought to play a role in adsorption and orientation of the biomolecules on the c/sNPs [2].

Successful immunomagnetic extraction of *B. cereus* and *E. coli* O157:H7 cells was confirmed by microbial plating. Table 1 shows the percent capture efficiencies obtained at several different original concentrations of each pathogen. Capture efficiency is calculated as log_10_ (*captured cell concentration*) divided by log_10 _ (*original cell concentration*). Since a 100 μL (0.1 mL) aliquot of each immuno-c/sNP-cell solution was applied to the SPCE, the estimated number of cells (CFU) actually present on the SPCE during electrochemical detection can be estimated by dividing the captured cell concentration (CFU/mL) by ten. These values are also given in Table 1 for each original cell concentration. The final column in Table 1 reports ΔQ values obtained during cyclic voltammetry of each test solution. Each reported value in Table 1 is the mean of three identical trials ± one standard deviation.

**Table 1:** Capture of *B. cereus* and *E. coli* O157:H7 cells by immuno-c/sNPs.

Original Est. Viable Cell Concentration (CFU/mL)	Mean Captured Viable Cell Concentration (CFU/mL)	Mean Capture Efficiency (log( *captured*)/log(*original*)) ± S.D.	Mean Est. Viable Cell Number (CFU) on SPCE	Mean Charge Transfer ΔQ (mC)
	± S.D. ( *n* = 3)	( *n* = 3)	± S.D. ( *n* = 3)	± S.D. ( *n* = 3)
*B. cereus*				
0 (Blank)	0	---	0	1.4 ± 0.20
4 × 10^0^	(0 ± 0) × 10^0^	(0 ± 0)%	0 ± 0	1.1 ± 0.15
4 × 10^1^	(1 ± 1) × 10^1^	(48 ± 43)%	1 ± 1	1.1 ± 0.03
3.9 × 10^2^	(1.2 ± 0.31) × 10^2^	(80 ± 4.4)%	12 ± 3.1	1.0 ± 0.06
*E. coli* O157:H7				
0 (Blank)	0	---	0	1.5 ± 0.07
6 × 10^0^	(7 ± 6) × 10^0^	(86 ± 75)%	0.7 ± 0.6	1.2 ± 0.12
6 × 10^1^	(1 ± 0.6) × 10^1^	(62 ± 9.8)%	1 ± 0.6	0.94 ± 0.03
5.9 × 10^2^	(1.7 ± 0.12) × 10^2^	(80 ± 1.1)%	17 ± 1.2	0.97 ± 0.26
5.9 × 10^3^	(1.6 ± 0.81) × 10^3^	(84 ± 7.3)%	160 ± 81	2.6 ± 0.51
5.9 × 10^4^	(1.3 ± 0.36) × 10^4^	(86 ± 2.4)%	1,300 ± 360	2.2 ± 0.37

Table 1 indicates that capture efficiency ranges from about 50 to 80% for *B. cereus* and 60 to 90% for *E. coli* O157:H7. The only exception to this is at the very lowest original cell concentration. In the case of *B. cereus*, no cells were captured (at least, as indicated by plate counts) at an original concentration of 4 × 10^0^ CFU/mL. However since the original cell concentration was so low, it is possible that 50% (2 CFU/mL) or more of the cells were indeed captured but were not present in the aliquot plated. The non-homogenous distribution of cells leads to greater uncertainty (high standard deviations) in reporting capture efficiency at very low pathogen concentrations. While the mean ΔQ value observed at this *B. cereus* concentration is not statistically different from the mean ΔQ value of the blank test, it is somewhat lower than the blank value, suggesting that in at least one of the three identical trials, a cell (or cells) may have indeed been captured and applied to the SPCE for detection. The concentration of *B. cereus* one order of magnitude greater (4 × 10^1^ CFU/mL) however, does result in a ΔQ value significantly different from the blank test, indicating a detection limit of 40 CFU/mL of *B. cereus*, which corresponds to approximately 1 CFU (estimated) present on the SPCE sensor. 

In the case of *E. coli* O157:H7, the greatest uncertainty (highest standard deviation) in capture efficiency is again observed at the lowest cell concentration (6 × 10^0^ CFU/mL), due to non-homogeneity of the sample. The mean ΔQ value observed this concentration is statistically different from the mean ΔQ value of the blank test. This indicates a detection limit of 6 CFU/mL of *E. coli* O157:H7, which corresponds to approximately 1 CFU (estimated) present on the SPCE sensor. 

The issues of uncertainty in capture efficiency and detection limit arise at low cell concentrations because only a small volume (100 µL) of the test solution is applied to either the culture plate or the SPCE sensor for electrochemical detection. For practical purposes, however, any unknown sample should be both diluted and concentrated (via IMS) by several orders of magnitude, and each of the resulting dilutions and concentrations tested independently. This will ensure that if a sample is positive, at least one of the dilutions or concentrations of the original sample will fall within the range of detectable concentrations, and yield a positive reading in the electrochemical detection step. Any presumptive positive sample would undergo further analysis to confirm the result. 

Improving the capture efficiency is an important future objective for optimization of this biosensing method. More efficient IMS capture would allow for more sensitive detection. One potential way to achieve better capture efficiency is to alter the method of immobilizing antibodies onto c/sNPs. Uniform orientation of antibodies with the constant (Fc) region bound to the nanoparticle surface and the antigen-binding (Fab) region outward can be achieved via biotin-avidin coupling [13,37] or Protein A-/G-mediated immobilization [38,39,40].

### 3.4. Electrochemical Detection of Bacterial Target

**Figure 4:**
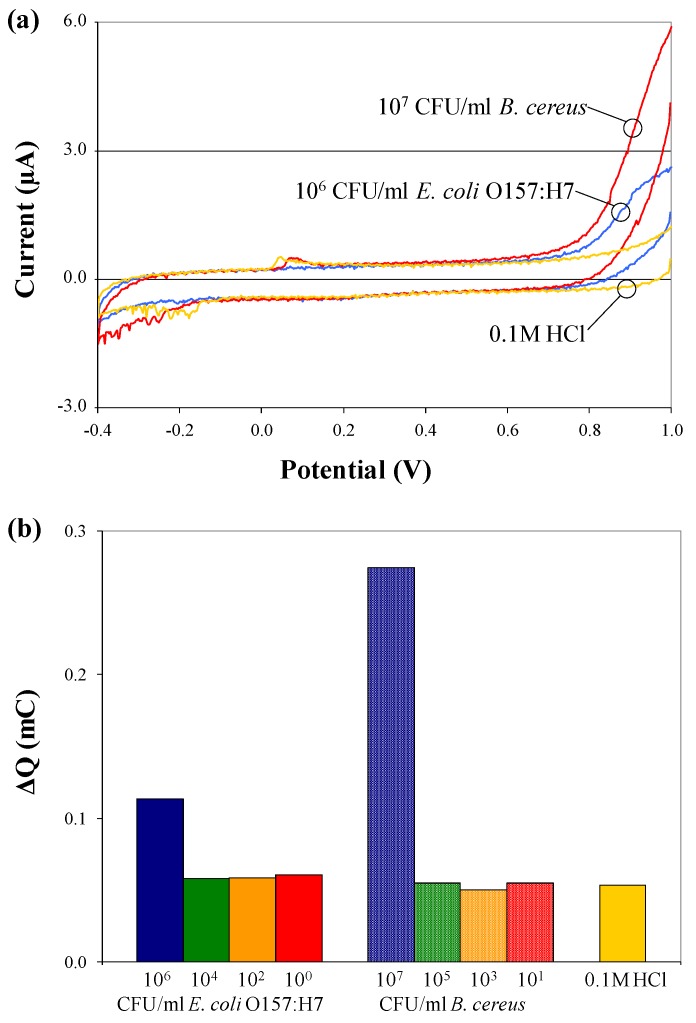
Cyclic voltammograms (**a**) and charge transfer values; (**b**) for electrochemical tests performed on pure *B. cereus* and *E. coli* O157:H7 cells, suspended at various concentrations in 0.1 M HCl solution, in the absence of c/sNPs.

From each electrochemical test, cyclic voltammograms (plot of response current *vs*. applied potential) were recorded. The third scan (of three consecutive scans performed) was chosen for analysis because it shows the most pronounced differences in current flow for different samples. Figure 4 depicts cyclic voltammograms and charge transfer values for pure (a) *B. cereus* and (b) *E. coli* O157:H7 cells suspended in 0.1 M HCl, in the absence of c/sNPs. The presence of cells has no effect on the electrochemical response, except at the highest concentrations tested, which are 10^6^ and 10^7^ CFU/mL. The increase in charge transfer observed at this high concentration can be explained by the conductivity of bacterial cell membranes and cytoplasm [19]. If the cells are packed densely enough, their membranes could form a conductive wire through which current is able to flow. Additionally, leakage of ions and cytoplasm out of the cells into the surrounding medium, which could be significant at high cell concentrations, increases the conductivity of the bulk solution and thereby increases current flow.

Although a low concentration of *B. cereus* or *E. coli* O157:H7 cells on the sensor surface in PBS buffer or HCl solution does not significantly influence the current response, a small number of cells complexed with c/sNPs and in the presence of excess (uncomplexed) c/sNPs has a much more pronounced effect on current flow. The conductivity of intact bacterial cells is on the order of 10^−3^ to 10^−2^ S/cm [41], whereas the conductivity of the electroactive c/sNPs is 3.3 S/cm [2]. Therefore when cells are bound to and dispersed in c/sNPs, they will function as barriers to current flow. Additionally, an external magnetic field is used to draw c/sNPs to the sensor surface and position cells in a layer directly above the surface (Figure 2), where the electric field is most concentrated [42]. In this location, the cells create the greatest obstruction to current flow. 

**Figure 5:**
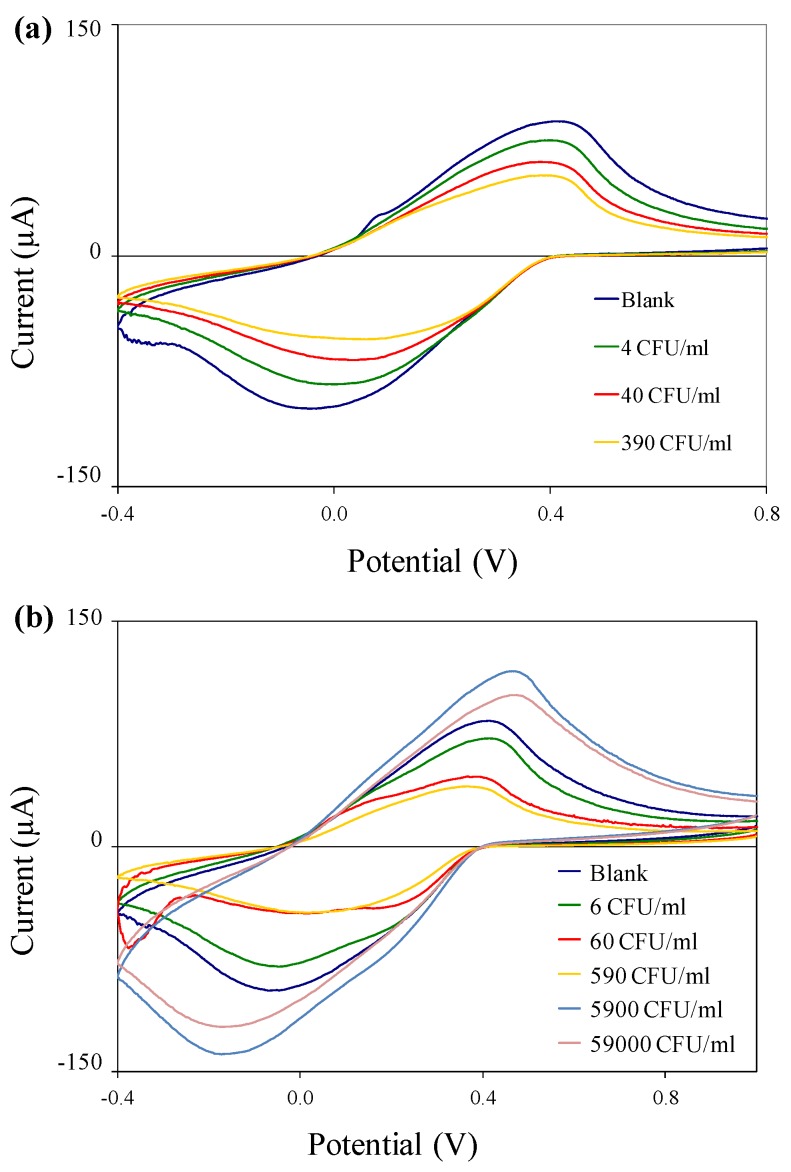
Cyclic voltammograms of immuno-c/sNP-cell solutions: (**a**) *B. cereus* cell concentrations ranging from 4 to 3.9 × 10^2^ CFU/mL; and (**b**) *E. coli* O157:H7 cell concentrations ranging from 6 CFU/mL to 5.9 × 10^4^ CFU/mL.

Electrochemical tests were performed on immuno-c/sNP-cell solutions with various concentrations of *B. cereus* and *E. coli* O157:H7 cells. The concentration of c/sNPs was constant at 1 mg/mL. In all cases, the polyaniline shell of the c/sNPs had been doped prior to testing by incubation in 0.1 M HCl. Figure 5 shows the cyclic voltammograms for (a) *B. cereus* and (b) *E. coli* O157:H7, comparing blank tests (no cells) to cell concentrations ranging from 10^0^ to 10^2^ (*B. cereus*) or 10^4^ (*E. coli* O157:H7) CFU/mL. It is evident that the current response decreases with an increasing number of cells present on the sensor, as expected. The only exception to this is at the two highest concentrations of *E. coli* O157:H7, 5.9 × 10^3^ and 5.9 × 10^4^ CFU/mL, which actually result in a current response much larger than that of the blank test.

**Figure 6:**
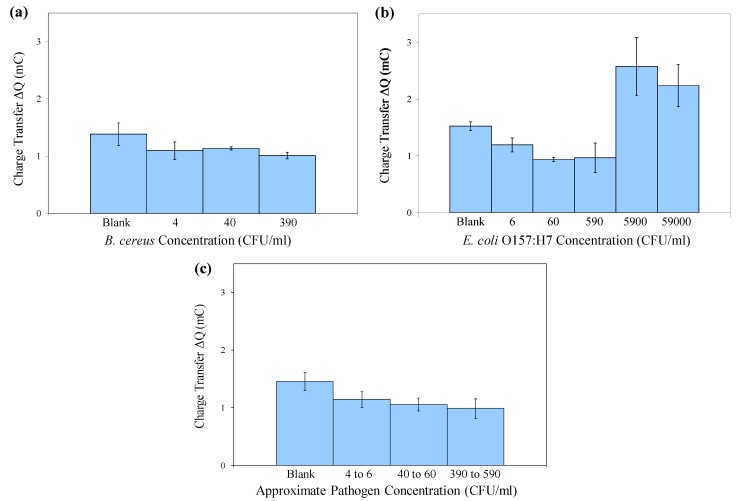
Mean charge transfer values obtained in cyclic voltammetry of immuno-c/sNP-cell solutions: (**a**) *B. cereus* cell concentrations ranging from 4 to 3.9 × 10^2^ CFU/mL (*n* = 3); (**b**) *E. coli* O157:H7 cell concentrations ranging from 6 CFU/mL to 5.9 × 10^4^ CFU/mL (*n* = 3); and (**c**) *B. cereus* and *E. coli* results displayed together (*n* = 6). Error bars represent ± one standard deviation.

Figure 6 shows the mean ΔQ values for the same experiment that was depicted in the cyclic voltammograms in Figure 5. Error bars represent ±1 standard deviation (*n* = 3). For both bacteria, the differences in ΔQ values between concentration groups are statistically significant with 95% confidence (p = 0.0356 for *B. cereus* and p = 0.0001 for *E. coli* O157:H7) by a single-factor analysis of variance (ANOVA).

For *E. coli* O157:H7, the system shows an upper detection limit falling between 5.9 × 10^2^ and 5.9 × 10^3^ CFU/mL. Further experiments would be required to determine the upper detection limit for *B. cereus*. The larger current responses observed at high cell concentrations (5.9 × 10^3^ and 5.9 × 10^4^ CFU/mL of *E. coli* O157:H7) indicate a Hook effect, a phenomenon commonly observed in immunoassays, in which the presence of analyte above a certain saturation concentration results in negative test readings. In this case, the increase in current flow at high cell concentrations is probably associated with conductance through the bacterial cell membranes and cytoplasm [19], and cell rupture or leakage, releasing ions into the bulk solution and thereby increasing its conductivity. It is important to note that for applications in biodefense, bacterial pathogens intentionally released as biological weapons would most likely be present at very high concentrations. The Hook effect can be unmasked by diluting the sample, thus it is critical in practical applications to test several dilutions (differing in concentration by orders of magnitude) of any unknown sample, to ensure that at least one dilution of a positive sample will fall within the range of detectable concentrations, so that the sample is flagged for further analysis.

Although the signals produced by low concentrations of cells are not distinguishable from one another (*i.e.*, the assay is not quantitative), they are statistically different from the blank signal. The data displayed in Figure 6(a,b) demonstrate that in pure culture, levels as low as 40 CFU/mL *B. cereus* and 6 CFU/mL *E. coli* O157:H7 are statistically different from the blank. When capture efficiency and solution volume are taken into consideration, these original concentrations may correspond to as few as 1 CFU present on the SPCE sensor during detection (Table 1). Therefore this is a rapid, inexpensive method for qualitatively screening food, water, or environmental samples for dangerous pathogens even at trace levels. In the case of *E. coli* O157:H7 or *Bacillus anthracis* (for which *B. cereus* is a surrogate), the presence of these pathogens at any level is unacceptable. After initial screening, any presumptive positive samples could be confirmed and quantified by traditional methods. 

Figure 6(c) displays the combined data for both *B. cereus* and *E. coli* O157:H7, collected on two separate days, indicating that the method is reproducible from day to day, using either of these two pathogens.

## 4. Conclusions

A novel electrochemical detection technique has been presented and applied to *B. cereus* and *E. coli* O157:H7, with lower detection limits of 40 CFU/mL and 6 CFU/mL, respectively, from pure culture, when pathogen concentration is in the range of 10^0^ to 10^2^ CFU/mL. (Samples containing pathogen concentrations greater than 10^3^ CFU/mL have generated false negative signals, and must undergo dilution prior to detection for accurate results). Cyclic voltammetry is combined with immunomagnetic separation in a rapid method requiring only 40 min for target recovery (and if necessary, concentration) and 25 min for detection. The method can be easily adapted for different bacterial targets simply by use of a different antibody, and the c/sNP-based magnetic extraction of the target can be performed in a variety of sample matrices [2]. Future research will include pathogen extraction and detection from food and environmental matrices, specificity tests, and validation of the sensor’s performance under non-laboratory environmental conditions. The entire system can be made portable through the use of a handheld potentiostat and laptop PC or PDA. The SPCE sensors employed as a detection platform require no surface modification, and are stable for long term storage (up to one year) at room temperature. The cost of the assay is not prohibitive, at approximately $1 per disposable SPCE sensor and $0.20 per test for reagents. Applications for this detection system include routine monitoring or emergency detection of bacterial pathogens for biodefense and biosecurity, or water and food safety.
